# Supplementary material for: Splice-Junction-Based Mapping of Alternative Isoforms in the Human Proteome
Source: Cell Rep. Author manuscript; Available in PMC 2020 Jan 15. (PMC6961840; doi:10.1016/j.celrep.2019.11.026)
Supplement: 2 [file NIHMS1546469-supplement-2.zip › DF1/PXD006675/PulmonaryVein/PulmonaryVein_8_MYBPC3_AITQLLCETEGR.pdf]

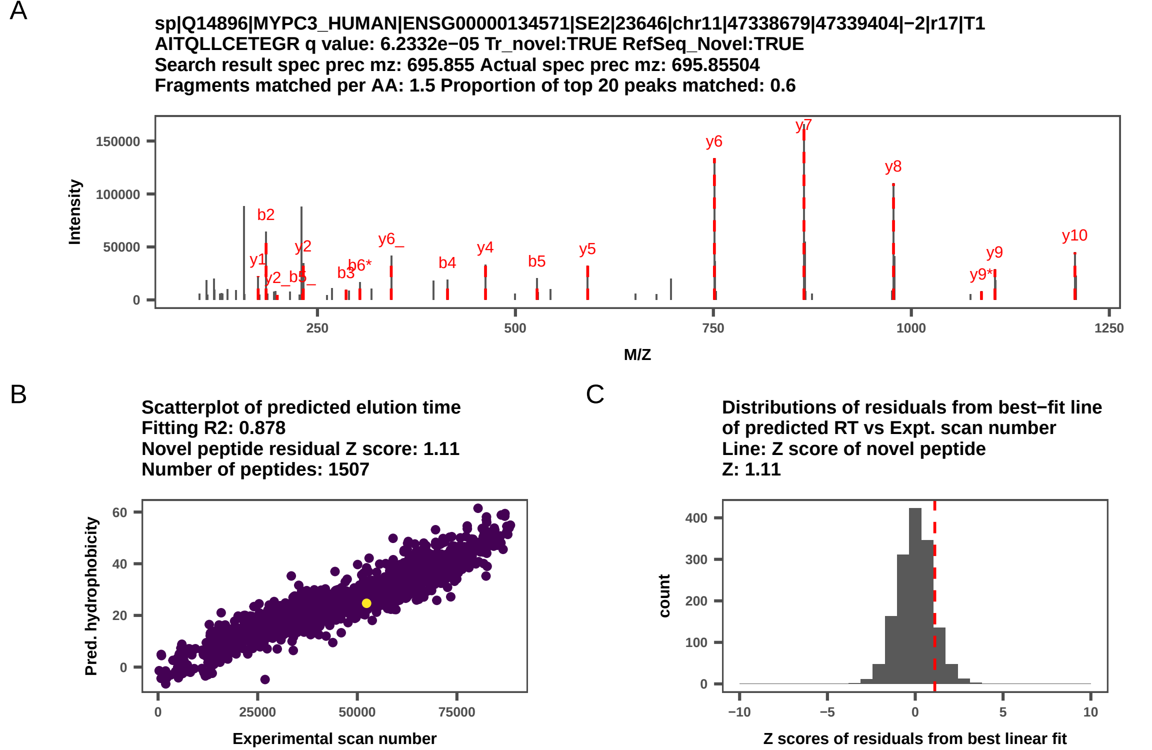
A
sp|Q14896|MYPC3_HUMAN|ENSG00000134571|SE2|23646|chr11|47338679|47339404|−2|r17|T1
AITQLLCETEGR q value: 6.2332e−05 Tr_novel:TRUE RefSeq_Novel:TRUE
Search result spec prec mz: 695.855 Actual spec prec mz: 695.85504
Fragments matched per AA: 1.5 Proportion of top 20 peaks matched: 0.6
Intensity
M/Z
B
Scatterplot of predicted elution time
Fitting R2: 0.878
Novel peptide residual Z score: 1.11
Number of peptides: 1507
Pred. hydrophobicity
Experimental scan number
C
Distributions of residuals from best−fit line
of predicted RT vs Expt. scan number
Line: Z score of novel peptide
Z: 1.11
count
Z scores of residuals from best linear fit
